# The extreme halophilic archaeon *Haloferax volcanii* reduced Fe(III) to conserve energy from extracellular electron transfer

**DOI:** 10.1128/jb.00345-25

**Published:** 2026-03-30

**Authors:** Yijing Miao, Fan Yang, Jian Yu, Pin Peng, Feng Zhao

**Affiliations:** 1 State Key Laboratory of Advanced Environmental Technology, Institute of Urban Environment, Chinese Academy of Sciences 85406 , Xiamen, Fujian, China; 2 University of Chinese Academy of Sciences 74519, Beijing, China; University of Virginia School of Medicine, Charlottesville, Virginia, USA

**Keywords:** archaea, extracellular electron transfer, hypersaline, dissimilatory Fe(III) reduction, biogeochemical cycle of iron

## Abstract

Iron availability affects oceanic primary productivity and the strength of the biological carbon pump. However, the mechanism of iron solubility in hypersaline environments is poorly understood. In this work, we investigated the extremely halophilic archaeon *Haloferax volcanii* to reduce ferrihydrite under 25% salinity, aiming to elucidate its energy metabolism associated with extracellular electron transfer (EET). Ferrihydrite reduction by *H. volcanii* reached a plateau around the eighth day, with an Fe(III) conversion of approximately 41%. The redox-active substances on the cell surface play a central role via EET. Proteomic analysis revealed that the relative abundance of membrane-bound quinoline *b*-type cytochrome oxidases (A0A841HB82, A0A841HBL7) increased by 73.8% and 120%, respectively, when *H. volcanii* used ferrihydrite rather than O_2_ as the terminal electron acceptor. Moreover, *H. volcanii* was found to secrete riboflavin, which can function as an electron shuttle mediating indirect EET. Our findings provide insights into the survival and evolutionary strategies of early life in anaerobic environments on Earth, and the EET capability of *H. volcanii* highlights its potential applicability in the treatment of pollutants in hypersaline wastewater.

**IMPORTANCE:**

As global warming intensifies, increased evaporation and seawater intrusion are leading to the expansion of high-salinity environments. Dissolved iron in these environments plays a crucial role in regulating the biogeochemical carbon cycle, thereby influencing global carbon dynamics. Microbial dissimilatory Fe(III) reduction may be the primary process driving the biogeochemical cycling of iron in such hypersaline environments. This study demonstrates that the extreme halophile *Haloferax volcanii* reduces ferrihydrite under high-salinity, anaerobic conditions. This reduction occurs primarily via membrane-associated cytochromes and secreted riboflavin. Consequently, investigating the extracellular electron transfer (EET) capabilities of halophilic archaea may provide new insights into the origin and maintenance of bioavailable iron under high-salinity stress.

## INTRODUCTION

Saline and hypersaline ecosystems ranging from coastal marshes and inland salt lakes to solar salterns represent globally significant repositories of carbon and trace metals (1–3). Their total water volume is comparable to that of Earth’s freshwater lakes (4), and as global warming intensifies, increased evaporation and seawater intrusion are creating more and more high-salinity environments (5). Within these systems, microbial reduction of Fe(III) serves critical functions. Specifically, it represents a key mechanism for anaerobic respiration, in which Fe(III) serves as a terminal electron acceptor to support microbial energy conservation. Additionally, iron cycling plays broader roles, including regulating microbial activity and influencing biogeochemical processes such as carbon mineralization and sequestration (6, 7). In sedimentary environments like marine sediments, salt lakes, and salt marshes, iron predominantly exists in insoluble Fe(III) forms, such as oxides and hydroxides, constituting a significant fraction of mineral phases (1, 8–10). However, the extremely low solubility of these Fe(III) minerals in high-salinity environments makes them difficult for organisms to utilize (11–13). This raises a fundamental biogeochemical question: what are the sources and mechanisms responsible for generating bioavailable (dissolved) iron to sustain microbial Fe(II) requirement in hypersaline systems?

In anoxic environments, the reduction of insoluble Fe(III) minerals typically requires strategies that couple cellular metabolism to solid-phase electron acceptors, most notably via extracellular electron transfer (EET) (14). This process is well characterized in freshwater and marine bacteria, such as *Geobacter* and *Shewanella*, where EET enables cells to respire on insoluble mineral oxides (15, 16). In contrast, our understanding of Fe(III) reduction and EET in hypersaline environments remains fragmentary (17). High salinity and osmotic stress drastically constrain microbial diversity, leading to a dominance of halophilic archaea (18, 19). Yet, little is known about how these extremophiles conserve energy in the absence of oxygen or how they interact with solid-phase electron acceptors such as Fe(III) oxides (20–22). Recent studies have revealed that some putative *c*-type cytochromes are potentially involved in EET of thermophilic archaea. Notably, the ten-heme cytochrome *c* (GAH_01256) from *Geoglobus ahangari* exhibits structural homology to the four-heme cytochrome *c* CymA in *Shewanella oneidensis* MR-1, a key component in Fe(III) oxide reduction via EET (23–26). This homology suggests GAH_01256 may similarly facilitate Fe(III) oxide reduction in *G. ahangari*. Additional cytochromes c (e.g., GAH_01306, GAH_00286, GAH_01534, GAH_01253) may also contribute to electron transport pathways mediating electron transfer from the cytoplasmic quinone pool to the extracellular space (25). Similarly, Smith et al. reported a greater abundance of cytochrome *c* in *Ferroglobus placidus* compared to other thermophilic Fe(III)-reducing archaea. Proteomic and transcriptomic analyses identified three multi-heme *c*-type cytochromes (MHCs) (Ferp_0670, Ferp_0672, Ferp_1267) as essential for Fe(III) reduction in this archaeon. These cytochromes exhibit genetic homology to electron transport proteins in *Geobacter* species (21, 27, 28). Cai et al. demonstrated that the anaerobic methanotrophic archaea “*Candidatus* Methanoperedens ferrireducens” can rely on Fe(III) reduction for anaerobic methane oxidation. Metatranscriptomic analysis revealed upregulation of genes encoding cytochrome *c* during this process, suggesting potential involvement of MHCs (29). Supporting this, transcriptomic studies identified the transmembrane polyheme cytochrome MmcA as a critical component in the EET of *Methanosarcina acetivorans* (30). Despite these advances, it remains unclear whether archaea can utilize EET mechanisms for dissimilatory Fe(III) reduction in hypersaline environments.

To address this gap, we investigated the model extreme halophile *Haloferax volcanii*, a genetically tractable archaeon that thrives in saturated salt environments, such as the Dead Sea (31, 32). Using ferrihydrite and conductive electrodes as extracellular electron acceptors, we examined whether *H. volcanii* is capable of reducing Fe(III) and sustaining physiological metabolic activity under anaerobic conditions. By integrating electrochemical analysis, liquid chromatography, and proteomics, the study revealed the EET pathway in *H. volcanii* and the pathway-related proteins. The results not only demonstrate a previously unrecognized capacity for dissimilatory Fe(III) reduction in an extreme halophile but also reveal unique strategies by which archaea may acquire and utilize dissolved iron in hypersaline environments. These findings provide new insights into the origin of bioavailable iron under high-salinity stress.

## MATERIALS AND METHODS

### Culture of *Haloferax volcanii*

*Haloferax volcanii* DSM 3757 used in this study was obtained from the German Collection of Microorganisms and Cell Cultures. The composition of the high-salt synthetic medium (SM) used was as follows (per liter) (33): 0.14 g of K_2_HPO_4_, 2.00 g of KCl, 0.54 g NH_4_Cl, 20.00 g of MgSO_4_ × 7 H_2_O, 250.00 g of NaCl, 1.00 g of CaCl_2_, 1.40 g of sodium L-lactate, 3.02 g of PIPES, 1.00 mL of trace element solution, 1.00 mL of vitamin solution. The detailed compositions of the trace element and vitamin solutions are provided in Tables 1 and 2. The pH of the culture medium was adjusted to 7.4–7.6 using NaOH. Cultures were incubated at 37°C with shaking at 150 rpm.

**TABLE 1:** Table of trace element solution SL-10 composition for *H. volcanii* SM medium

Name	Quantity	Unit
HCl (25%)	10.00	mL
FeCl_2_ × 4 H_2_O	1.50	g
ZnCl_2_	70.00	mg
MnCl_2_ × 4 H_2_O	100.00	mg
H_3_BO_3_	6.00	mg
CoCl_2_ × 6 H_2_O	190.00	mg
CuCl_2_ × 2 H_2_O	2.00	mg
NiCl_2_ × 6 H_2_O	24.00	mg
Na_2_MoO_4_ × 2 H_2_O	36.00	mg
Distilled water	990.00	mL

**TABLE 2:** Table of composition of vitamin solution for *H. volcanii* SM medium

Name	Quantity	Unit
Vitamins B12	100.00	mg
Para-aminobenzoic acid	80.00	mg
D- (+) biotin	20.00	mg
Niacin (vitamin B3)	200.00	mg
Calcium pantothenate	100.00	mg
Vitamins B6	300.00	mg
Vitamins B1	200.00	mg
Distilled water	1,000.00	mL

### Fe(III) reduction assay

*Haloferax volcanii* was inoculated at a rate of 5% into 100 mL of fresh high-salt medium, and incubated at 37°C with shaking at 150 rpm for approximately 7 days until the mid-late logarithmic growth phase (OD_600_ ≈ 1.5). Prior to sterilization, 50 mL of sterile SM medium was subjected to a multi-step deoxygenation process using high-purity N_2_ (99.99%). The effectiveness of this procedure was verified in preliminary experiments employing resazurin as an oxygen indicator. The deoxygenation steps were as follows: (i) sparging the liquid phase for 30 min via a gas needle submerged below the liquid surface; (ii) sealing the bottle with a butyl rubber stopper and continuing N_2_ sparging below the liquid surface for a further 30 min; (iii) raising the gas needle above the liquid level and flushing the headspace with N_2_ for an additional 30 min. Following deoxygenation, the bottle was immediately sealed tightly with an aluminum crimp seal, and the medium was sterilized by autoclaving at 121°C for 20 min. *H. volcanii* cells were harvested by centrifugation and washed twice with SM medium lacking lactate. The washed cell pellet was then resuspended in the prepared 50 mL of sterile, deoxygenated SM medium (prepared as above). Ferrihydrite was synthesized following previously reported procedures (34, 35) and added to the cell suspension at a final concentration of 3 mM Fe-total. For riboflavin validation experiments, 0.2 μM riboflavin was added to examine its potential facilitation of the indirect EET mechanism. All experimental setups were performed in triplicate to ensure reproducibility and reliability.

### Chemical measurements

Samples were immediately mixed with an equal volume of 12 M HCl upon collection. After complete dissolution, five volumes of ultrapure water were added for Fe detection. The concentrations of Fe-total and Fe(II) were quantified using the phenanthroline method (36). For pH, lactate, and riboflavin measurement, samples were centrifuged at 10,000 × *g* for 5 min, and the supernatant was collected. The remaining precipitate was used to determine the total protein content by the bicinchoninic acid (BCA) assay.

After completion of the ferrihydrite reduction experiment, the cell-free supernatant was collected for lactate and riboflavin quantification. Lactate was analyzed by high-performance liquid chromatography (HPLC, Shimadzu LC-20A, Japan) equipped with an SPD-M40 detector and an Aminex HPX-87h column (Bio-Rad, 300 × 7.8 mm, USA). The mobile phase consisted of 4 mM H_2_SO_4_, operated at a flow rate of 0.6 mL/min at 35°C with a 20 µL injection volume. Riboflavin was detected by HPLC (Shimadzu LC-20A, Japan) equipped with an RF-20A detector and a C18 column (DAISOPAK, 4.6 mm I.D. × 150 mm, Japan). A 20 μL sample was automatically injected into the HPLC system, and glacial acetic acid:methanol:water (vol:vol:vol = 1:30:69) was used as mobile phase A, while methanol served as mobile phase B. The HPLC was run at a flow rate of 0.5 mL/min at 40°C. The excitation and emission wavelengths were set at 420 and 530 nm, respectively. Gradient elution was performed following a previously published protocol (37) (see Table 3).

**TABLE 3:** Gradient elution procedure for lactate measurement in HPLC

Time (min)	Mobile phase A	Mobile phase B
0.0	85	15
5.0	85	15
6.0	82	18
9.0	65	35
12.0	65	35

### Proteomic analysis

#### Protein extraction and analysis

Protein analysis was performed on biomass samples collected from Fe(III)-reducing incubation batches. Three vials were harvested as independent biological replicates. Each sample was lysed by adding four volumes of lysis buffer (8 M urea, 1% protease inhibitor, wt/vol) and subjected to ultrasonic disruption. The lysates were centrifuged at 12,000 × *g* for 10 min at 4°C, and the resulting supernatants were collected. Protein concentrations were determined using a BCA protein assay kit (Solarbio, China). Equal amounts of protein from each sample were used for enzymatic digestion. Trichloroacetic acid was added to a final concentration of 20%, and the mixture was vortexed and incubated for 2 h. The precipitated proteins were collected and washed 2–3 times with pre-cooled acetone. After air-drying the precipitate, proteins were resuspended in 200 mM triethylammonium bicarbonate, sonicated to disperse the pellet, and digested overnight with trypsin. Dithiothreitol was then added to a final concentration of 5 mM, followed by the addition of iodoacetamide. The mixture was incubated in the dark at room temperature. Finally, the peptides were analyzed by the EASY-nLC 1200 ultra-high performance liquid chromatography system (UPLC, Thermo Fisher Scientific, USA).

#### Proteome database search

The resulting MS/MS spectra were processed using the MaxQuant search engine (v.1.6.15.0). The enzyme specificity was set to Trypsin/P, allowing a maximum of one missed cleavage. Fixed modifications included N-terminal methionine excision and carbamidomethylation of cysteine residues. Deep learning algorithms were used to construct theoretical spectral libraries, and inverse libraries were added to calculate the false positive rate due to random matching.

#### Protein functional annotation

To gain a comprehensive understanding of the functional characteristics of the identified proteins, detailed functional annotation was performed. The analyses included identification of protein domains, KEGG pathway mapping, and prediction of subcellular localization. Protein domain annotation was conducted using the Pfam database and the corresponding PfamScan tool. KEGG pathway annotation was performed to assign potential biological functions. Subcellular localization of the identified proteins was predicted using the WoLF PSORT software.

#### Differential protein screening

Fisher’s exact test was employed to evaluate the statistical significance of functional enrichment among differentially expressed proteins, using all identified proteins as the background. Functional categories with a fold change >1.5 and a *P* value <0.05 were considered statistically significant.

The mass spectrometry proteomics data have been deposited to the ProteomeXchange Consortium (https://proteomecentral.proteomexchange.org) via the iProX partner repository (38, 39) with the data set identifier PXD074223.

### Electrochemical measurements

Electrochemical activity was evaluated using a single-chamber, three+electrode system. *H. volcanii* immobilized on carbon-felt electrodes (2 × 2 cm^2^) served as the working electrode. A sterile carbon-felt electrode (2 × 2.5 cm^2^) was used as the counter electrode, and an Ag/AgCl electrode (saturated KCl) functioned as the reference electrode. The SM medium served as the electrolyte and was rendered anoxic by purging with high-purity N_2_ for 90 min. A potentiostat (CHI 1000B, Chenhua Instruments Co., Ltd., Shanghai, China) was employed to maintain the working electrode potential at 0.3 V. After incubation at constant potential, cyclic voltammetry was performed using a CHI 660D potentiostat (Chenhua Instruments Co., Ltd., Shanghai, China) at a scan rate of 0.01 V/s over a potential range of 0.4 to −0.6 V. All electrochemical tests were conducted in triplicate at 37°C.

### Statistical analysis

All statistical analyses were performed using SPSS Statistics 22.0 (IBM Corp., Armonk, NY, USA). Tukey’s test determined statistical significance among different setups. ****P < 0.0001; ***P < 0.001; **P < 0.01; *P < 0.05, and n.s., not significant.

## RESULTS

### *Haloferax volcanii* reduced Fe(III) oxides

**Fig 1:**
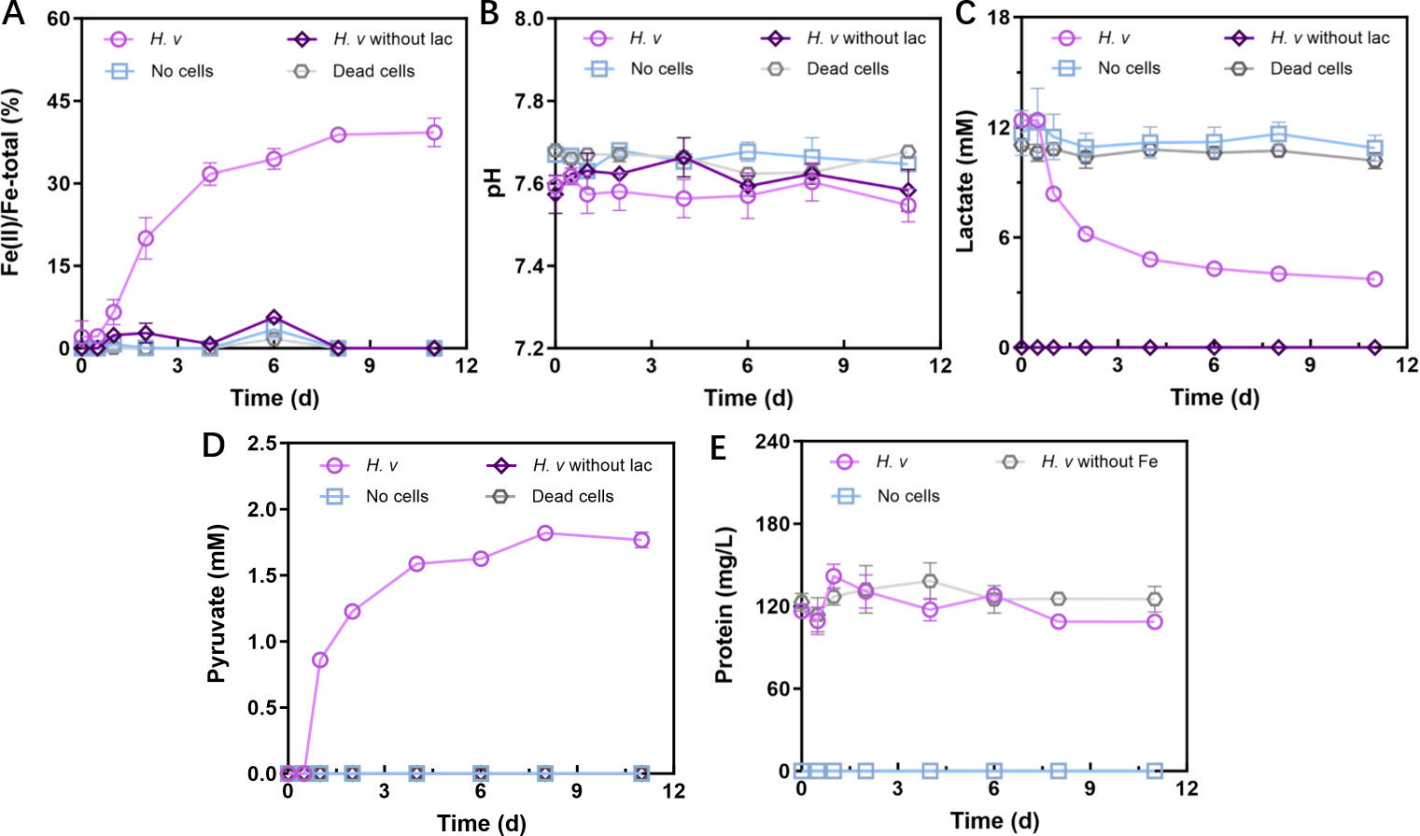
Significant reduction of Fe(III) oxides by *H. volcanii*. (**A**) The ratio of Fe(II)/Fe-total; (**B**) pH; (**C**) the concentration of lactate; (**D**) the concentration of pyruvate; (**E**) the concentration of protein. Error bars represent standard deviations from triplicate experiments.

**Fig 2:**
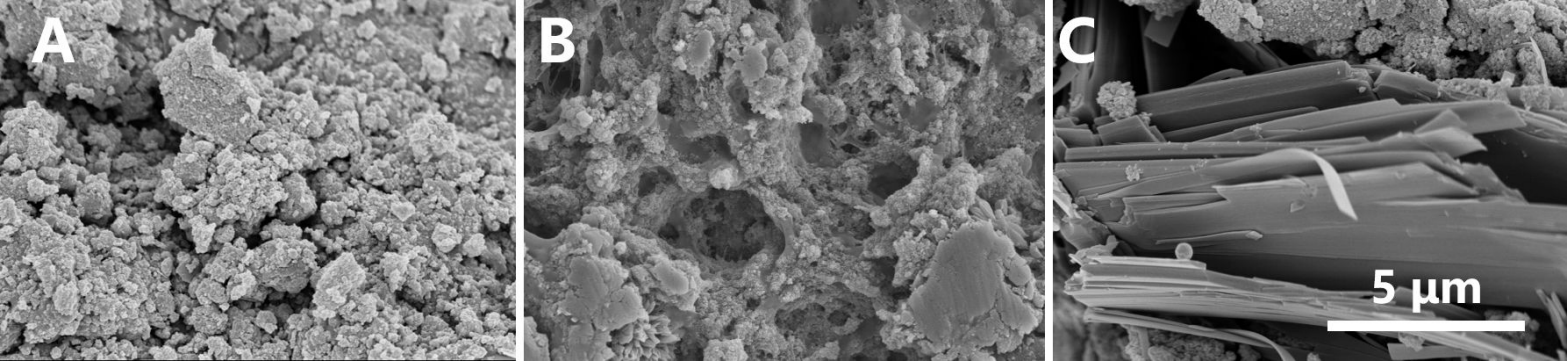
SEM characterization of Fe (III) reduction. (**A**) Minerals before reduction; (**B**) minerals reduced by dead cells of *H. volcanii*; (**C**) minerals reduced by *H. volcanii*.

To investigate the extracellular Fe(III) oxide reducing capability of *H. volcanii*, a batch experiment was established using lactate as the sole carbon source and electron donor. The incubation was carried out for a total of 11 days. During the first 8 days, the Fe(II) reduction rate in the *H. volcanii* group exhibited a progressive increase (Fig. 1A). By the end of the experiment, 1,138.67 ± 20.98 µM Fe(II) had accumulated in the system, corresponding to an overall reduction efficiency of approximately 41%. The average daily reduction rate was 103.52 ± 1.91 μM_Fe(II)_·day^-1^. Negligible Fe(III) reduction was observed in all control groups, including those lacking lactate (the electron donor), the sterile medium, and the dead cell suspension. These results demonstrate that the significant Fe(III) reduction by *H. volcanii* requires both viable cells and an available electron source. The minor Fe(III) reduction observed in sterile controls likely resulted from the intrinsic phase transformation of ferrihydrite. As a metastable mineral, ferrihydrite maintained high reactivity due to Ca^2+^/Mg^2+^, which delays its conversion to hematite/goethite. And may undergo limited abiotic reduction by trace reductants such as dissolved H_2_ (40–42). The reduced minerals were collected for scanning electron microscopy (SEM) analysis (Fig. 2). Before the reaction, ferrihydrite appeared amorphous and lacked discernible crystalline features. After reduction, the resulting minerals exhibited a distinctive rhombohedral, layered morphology, indicating structural transformation during the reduction process.

The pH of the *H. volcanii* culture remained stable at approximately 7.6 throughout the 11-day incubation period (Fig. 1B), showing no significant difference from the abiotic control (*P* = 0.16), thereby ruling out the possibility that iron reduction was driven by solution acidification. Changes in lactate concentration over the 11-day incubation in the *H. volcanii* treated group decreased markedly from 12.38 ± 0.05 mM to 3.73 ± 0.25 mM (Fig. 1C), whereas no measurable change occurred in the control group. These results indicate that *H. volcanii* actively utilized lactate as an electron donor to support its metabolic processes under the tested conditions. Pyruvate was the only extracellular metabolite detected in cell-free supernatants, accumulating to 1.77 ± 0.04 mM after 11 days (Fig. 1D). Based on the stoichiometry of lactate oxidation to pyruvate (CH_3_CHOHCOOH → CH_3_COCOOH + 2H^+^ + 2e^−^), this corresponds to the incomplete oxidation of approximately 1.77 mM lactate and the release of an estimated 3.54 mM electrons, of which 1.14 mM electrons are utilized for Fe(III) reduction. Collectively, these findings provide strong evidence that *H. volcanii* can transfer electrons to Fe(III) oxides and mediate their reduction under strictly anaerobic conditions (43). During the reduction process, the biomass of *H. volcanii* exhibited no significant increase by the end of the experiment (*P* = 0.12), as assessed based on protein content (Fig. 1E). This observation suggests that Fe(III) reduction served primarily as a physiological strategy for maintaining basic physiological metabolic activity in *H. volcanii*, rather than promoting substantial biomass accumulation.

### The extracellular electron transfer mechanism of *H. volcanii*

#### Characterization of redox activity based on electrochemical testing

**Fig 3:**
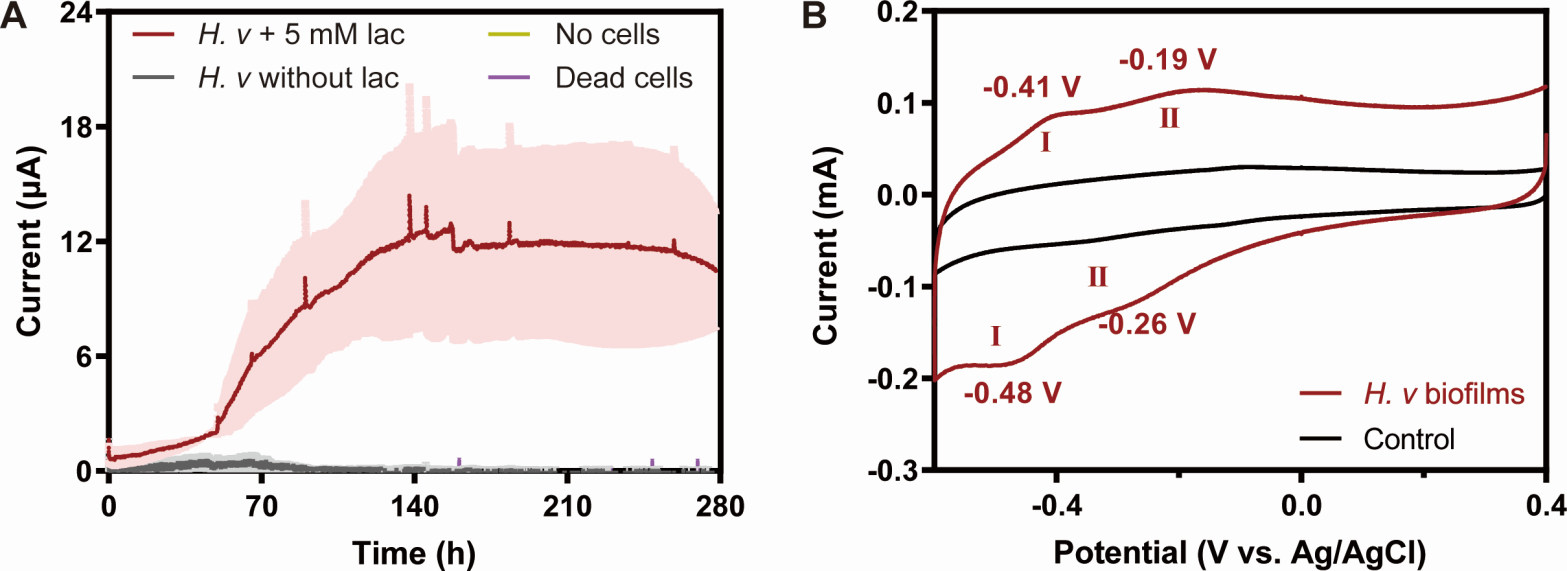
Electrochemical characterization of *H. volcanii*. (**A**) Time-current variation; (**B**) cyclic voltammetry curves of biofilms.

To further elucidate the EET mechanism of *H. volcanii*, electrochemical measurements were performed using electrodes instead of Fe(III) oxides as the electron acceptor. As shown in Fig. 3A, when a potential of 0.3 V (vs Ag/AgCl) was applied, the current gradually increased and reached approximately 13 µA after 156 h, followed by a subsequent decline. Previous research has shown that electroactive bacteria inoculated into electrochemical reactors typically exhibit a peak current after a period of adaptation. The current arises from the transfer of electrons generated during microbial metabolism to the electrode. Accordingly, as microorganisms progressively colonize the electrode surface, the current increases and gradually stabilizes upon biofilm maturation (44, 45). However, as substrates are progressively depleted, the current subsequently declines (46). Post-experiment analyses indicated that lactate had been completely depleted, decreasing from 4.69 ± 0.28 mM to undetectable levels. This substrate exhaustion likely accounted for the subsequent current decline. In addition, the death or inactivation of cells within the inner layer of the biofilm may have further reduced the measurable current. Because the electrochemical reactors operated under static conditions without agitation, substrates in the vicinity of the electrodes were rapidly consumed and not replenished efficiently, which may also have contributed to the observed decline (47). Overall, these results demonstrate that *H. volcanii* is also capable of transferring electrons to electrodes and is an electrogenic microorganism.

To determine whether *H. volcanii* exhibits redox characteristics, a cyclic voltammetric test was performed to evaluate its electrochemical behavior on a biofilm-coated electrode. As shown in Fig. 3B, the *H. volcanii* biofilm displayed two distinct oxidation peaks at −0.41 and −0.19 V, and two corresponding reduction peaks at −0.48 and −0.26 V. The presence of distinct oxidation and reduction peaks demonstrates that the *H. volcanii* biofilm possesses redox-active substances on its surface. These substances, which exhibit redox activity at the observed potentials, are likely the primary mediators responsible for its EET capability.

#### Indirect EET analyzed by supernatant assay

**Fig 4:**
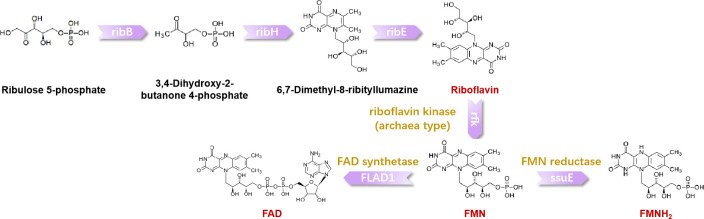
Potential riboflavin synthesis pathways in *H. volcanii* (48–50). Riboflavin biosynthesis utilizes ribulose-5-phosphate (R5P) as a precursor. R5P is converted to 3,4-dihydroxy-2-butanone-4-phosphate (DHBP) by DHBPP synthase (ribB). Subsequently, lumazine synthase (ribH) catalyzes the condensation of DHBP to form 6,7-dimethyl-8-ribityllumazine (DRL). Riboflavin synthase (ribE) then converts DRL into riboflavin. For metabolic utilization, riboflavin undergoes phosphorylation. Archaeal riboflavin kinase (RFK) utilizes ATP to convert riboflavin into flavin mononucleotide (FMN). Flavin adenine dinucleotide (FAD) synthase (FLAD1) then adenylates FMN to generate FAD. Finally, FMN reductase (ssuE) reduces both FMN and FAD to their hydroquinone forms, FMNH_2_ and FADH_2_, respectively.

**Fig 5:**
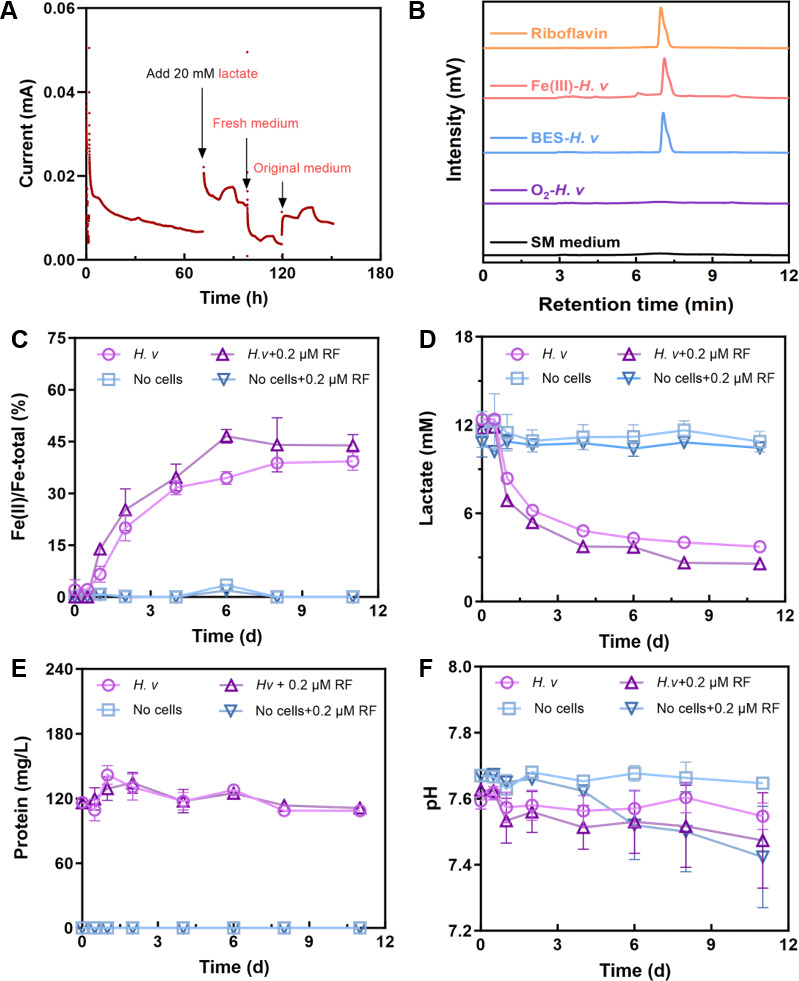
Evidence of riboflavin secretion by *H. volcanii*. (**A**) Current-time curves under different treatments; (**B**) liquid chromatograms of 0.4 μM riboflavin standard, supernatant of *H. volcanii* reduced Fe(III) oxides experimental group, supernatant of *H. volcanii* in bioelectrochemical systems, supernatant from aerobic culture of *H. volcanii* and blank medium; (**C**) the ratio of Fe(II)/Fe-total; (**D**) the concentration of lactate; (**E**) the concentration of protein; (**F**) pH. Error bars represent standard deviations from triplicate experiments. There was no significant increase in biomass and pH with or without riboflavin (*P* > 0.05).

Based on genomic data of *H. volcanii* in the NCBI database, it was identified that *H. volcanii* possesses genes related to riboflavin synthesis (Fig. 4). Riboflavin has been reported to mediate indirect EET processes (46). To further verify whether *H. volcanii* can secrete riboflavin as an electron mediator in this system, the experiment involving the replacement of the electrolyte supernatant was performed (Fig. 5A) (46). Briefly, *H. volcanii* cells were inoculated into an electrolyte solution (SM medium without lactate) that did not contain lactate and incubated under an applied potential of 0.3 V (vs Ag/AgCl) to induce a starvation phase, aiming to completely deplete any residual lactate. At 72 h, 20 mM lactate was added to the electrolyte, resulting in a slight increase in current. At 98 h, the electrolyte was replaced with a fresh solution, which caused a sharp drop in current. Subsequently, at 120 h, the original electrolyte was added back, and the current partially recovered and increased markedly. These observations indicate that the supernatant of *H. volcanii* likely contained a compound capable of mediating electron transfer. The collected supernatant from this stage, together with the supernatant obtained after ferrihydrite reduction, was analyzed by HPLC. A distinct peak appeared at approximately 6.98 min, matching the retention time of the riboflavin standard. In contrast, no such peak was observed when *H. volcanii* used O_2_ as the electron acceptor (Fig. 5B). This evidence demonstrates that when *H. volcanii* utilizes insoluble substances as extracellular electron acceptors, it can secrete riboflavin as an electron shuttle to mediate indirect electron transfer.

To evaluate the potential role of riboflavin as an electron shuttle facilitating EET to Fe(III) oxides, 0.2 µM riboflavin was added to the experimental group, while an unamended group was maintained as the control. As shown in Fig. 5C, after 6 days, cultures supplemented with riboflavin reduced 1,087 ± 89 µM Fe(III), corresponding to an overall reduction efficiency of ~45%, compared with 917 ± 50 µM (34%) in the control group. This represents a relative increase in the Fe(III) reduction rate of approximately 18%. In the presence of riboflavin addition, the average daily reduction rate reached 181.19 ± 14.90 μM_Fe(II)_·day^-1^, while the *H. volcanii*-only group exhibited 152.83 ± 8.40 μM_Fe(II)_·day^-1^. These findings clearly indicate that the presence of riboflavin enhanced Fe(III) oxide reduction. Similarly, the concentration of lactate as an electron donor reflected a similar trend (Fig. 5D). After 11 days, lactate utilization in the riboflavin-supplemented system reached 9.22 ± 0.04 mM, whereas that in the *H. volcanii* without riboflavin culture decreased by 8.65 ± 0.22 mM (*P* = 1.4 × 10^−6^). There was no significant increase in biomass and pH with or without riboflavin (*P* > 0.05, Fig. 5E and F). Taken together, the increase in Fe(II) concentration is attributed to the enhancement of EET by riboflavin. These results suggest that riboflavin-mediated indirect electron transfer serves as an important pathway enabling *H. volcanii* to reduce extracellular Fe(III) oxides.

### Differential protein abundance analysis based on proteomic results

**Fig 6:**
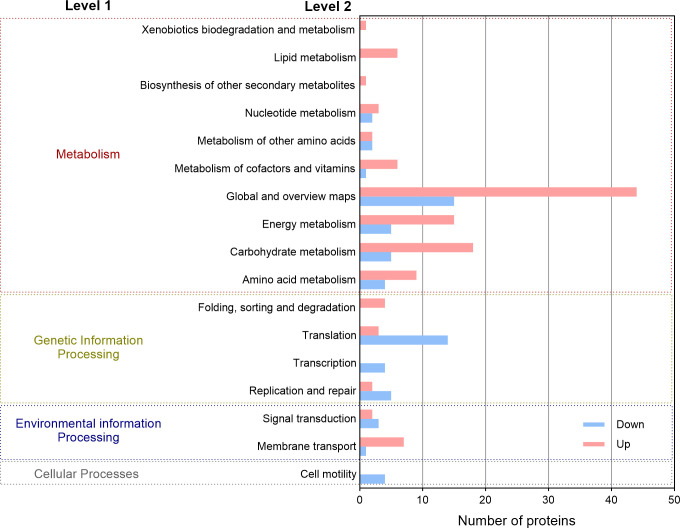
KEGG functional annotation of differently expressed proteins of *H. volcanii* under the condition of Fe(III) oxide as electron acceptor compared with the condition where O_2_ as the electron acceptor.

To further elucidate the EET mechanism, proteomic analysis was performed on *H. volcanii* using O_2_ as an electron acceptor and Fe(III) oxides as an electron donor, respectively. Proteins exhibiting significant changes in abundance were identified based on univariate statistical criteria (|log_2_(FC)| > 1.0 and *P* < 0.05). The results showed that a total of 254 proteins exhibited significant differences in relative abundance between conditions, including 137 proteins with increased relative abundance and 117 proteins with decreased relative abundance. When Fe(III) oxides served as the electron acceptor, proteins involved in energy production and conversion, amino acid metabolism and transport, and environmental information processing and genetic information processing were predominantly more abundant (Fig. 6). Among proteins showing increased relative abundance, 35.77% were predicted to be localized outside the cytoplasmic membrane, while 10.22% were associated with the plasma membrane. In contrast, most proteins showing decreased relative abundance were transcription factors that were more abundant under aerobic conditions. Additionally, two flagellar proteins (B9LTG4, B9LTG7) also showed decreased relative abundance (51); this downregulation with Fe(III) oxides may reflect an energy-conservation strategy, whereby *H. volcanii* reallocates cellular resources toward essential metabolic processes.

**Fig 7:**
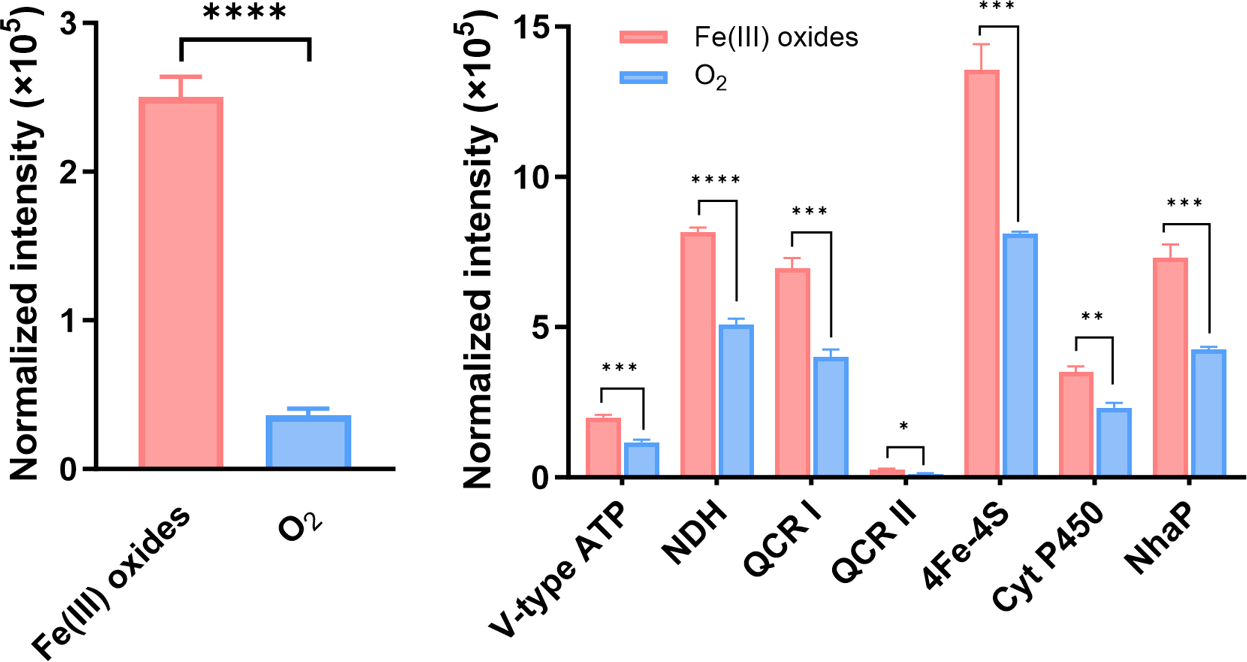
Differential expression of key proteins. (**A**) Normalized intensity of protein expression of cytochrome *c* (B9LN21) in cells cultured under conditions using Fe(III) oxide and O_2_ as electron acceptors; (**B**) normalized intensity of expression of key proteins other than cytochrome *c* associated with EET. V-type ATP: V-type ATP synthase subunit I. NDH: NADH: quinone oxidoreductase. QCR: quinol-cytochrome oxidoreductase complex cytochrome *b*. 4Fe-4S: 4Fe-4S ferredoxin-type domain-containing protein. Cyt P450: cytochrome P450. NhaP: NhaP-type Na^+^/H^+^ or K^+^/H^+^ antiporter. Error bars represent standard deviations from triplicate experiments. *** and **** indicate significant differences between the control and treatments at *P* < 0.001 and 0.0001, respectively.

Proteomic analysis revealed that *H. volcanii* contains multiple *c*-type cytochrome proteins (B9LSP0, B9LUQ8, B9LN21, B9LUQ9, detailed in Table 4). All four proteins correspond to monoheme cytochrome *c* subunit II (COX2) and are predicted to be localized in the cell membrane. Among these *c*-type cytochromes, the protein B9LN21 encoded by the Hlac_1165 gene comprises four subunits, and its expression under anaerobic conditions was 6.92-fold higher than that observed under aerobic conditions (Fig. 7A).

**TABLE 4:** Changes in relative abundance of *c*-type cytochrome proteins in *H. volcanii* cells^*a*^

Locus ID	Heme groups	Predicted localization	Gene name	Fold change in relative abundance [Fe(III) vs O_2_]	*P* value
B9LS12	2	Membrane	Hlac_2310 (menaquinol-cytochrome-*c* reductase)	NS	NS
B9LTW6	2	Membrane	Hlac_0648 (menaquinol-cytochrome-*c* reductase)	NS	NS
B9LSP0	1	Membrane	Hlac_2412	NS	NS
B9LUQ8	1	Membrane	Hlac_0785	NS	NS
B9LN21	1	Cytoplasmic	Hlac_1165	6.923897	1.59 × 10^−5^
B9LUQ9	1	Membrane	Hlac_0786	NS	NS

^*a*^ Shown are genes encoding electron transport proteins that are *c*-type cytochromes and that were differentially expressed in *H. volcanii* cells grown with lactate as the electron donor and either O_2_ or insoluble Fe(III) oxides as the electron acceptor. Proteins were only considered differentially expressed if the *P* value was ≤ 0.05. NS, no significant difference in read abundance between conditions.

In addition to cytochrome *c*, the relative abundance changes of other key proteins related to EET are displayed in Fig. 7B (detailed in Table 5). Notably, a cytochrome P450 (B9LUN1), which contains a heme cofactor essential for redox catalysis, showed a 51.1% increase in normalized relative abundance under Fe(III) oxide-reducing conditions. Likewise, the expression of a 4Fe-4S ferredoxin-type domain-containing protein (B9LT38) increased by 67.2%. Given their inherent roles as redox-active proteins involved in electron transfer in biological systems (52, 53), this pronounced upregulation specifically during Fe(III) oxides as the final electron acceptor strongly suggests their potential involvement as candidate electron carriers in the EET process of *H. volcanii*. The membrane-associated quinol-cytochrome oxidoreductase complex cytochrome *b* with two heme motifs (QCR I, A0A841HB82; and QCR II, A0A841HBL7) showed 73.8% and 120% increases in normalized relative abundance, respectively, which may be involved in electron transfer across the cytoplasmic membrane.

**TABLE 5:** Changes in relative abundance of proteins in *H. volcanii* other than *c*-type cytochromes that may be related to electron transport proteins^*a*^

Locus ID	Gene product	Gene name	Fold change in relative abundance [Fe(III) vs O_2_]	*P* value
B9LWX1	ABC transporter	Hlac_3452	2.87	1.54 × 10^−5^
B9LPL7	Copper-binding protein	Hlac_1722	2.62	8.40 × 10^−6^
B9LQL3	Binding-protein-dependent transport systems inner membrane component	Hlac_2056	2.94	1.41 × 10^−5^
B9LQL2	ABC transporter	Hlac_2055	3.13	7.52 × 10^−6^
B9LT09	Molybdate/tungstate import ATP-binding protein WtpC	Hlac_0472	1.64	6.05 × 10^−3^
B9LUX4	ABC transporter	Hlac_0852	2.71	9.57 × 10^−5^
B9LU73	Binding-protein-dependent transport systems inner membrane component	Hlac_2696	2.63	1.45 × 10^−6^
A0A841HA16	V-type ATP synthase subunit I	HNR49_000637	1.71	4.71 × 10^−4^
B9LS41	V-type ATP synthase alpha chain	atpA	1.62	5.08 × 10^−3^
A0A841HBA9	Aconitate hydratase	HNR49_000801	1.61	5.49 × 10^−3^
Q59455	Pyruvate ferredoxin oxidoreductase alpha subunit (Fragment)	HNR49_001572	1.88	3.81 × 10^−8^
B9LRY7	Acetamidase/formamidase	Hlac_2285	2.47	4.13 × 10^−3^
B9LMJ9	Acetate-CoA ligase	Hlac_0990	1.77	3.06 × 10^−4^
B9LN38	Methylmalonyl-CoA mutase, large subunit	Hlac_1182	1.74	0.0317
B9LV71	Aconitate hydratase 1	Hlac_3040	2.05	9.04 × 10^−4^
B9LNG0	Acetate-CoA ligase	Hlac_1306	1.95	3.50 × 10^−5^
B9LQT7	DoxX family protein	Hlac_0083	1.60	1.09 × 10^−5^
A0A841HAQ4	Glutamine synthetase	HNR49_000593	1.80	0.0395
A0A841HAH8	NADH-quinone oxidoreductase subunit C/D	HNR49_001350	1.61	4.12 × 10^−5^
B9LPU4	Cytochrome oxidase assembly	Hlac_1803	3.27	1.15 × 10^−3^
B9LMG3	DoxX family protein	Hlac_0953	12.6	5.16 × 10^−4^
B9LUZ6	Acetolactate synthase	Hlac_0875	1.61	4.00 × 10^−6^
B9LSM2	GTP-dependent dephospho-CoA kinase	Hlac_2394	1.77	0.0243
A0A841H7I2	Flavin-dependent thymidylate synthase	thyX	1.53	8.50 × 10^−4^
A0A841H836	Pyridoxal 5′-phosphate synthase subunit PdxS	pdxS	1.50	0.0239
B9LR55	Glutamyl-tRNA reductase	hemA	3.79	3.86 × 10^−5^
A0A841HB82	Quinol-cytochrome oxidoreductase complex cytochrome *b* subunit	HNR49_001302	1.74	2.95 × 10^−4^
A0A841HBL7	Quinol-cytochrome oxidoreductase complex cytochrome *b* subunit	HNR49_001303	2.20	0.0233
B9LT38	4Fe-4S ferredoxin-type domain-containing protein	Hlac_0501	1.67	1.57 × 10^−4^
Q59455	Pyruvate ferredoxin oxidoreductase alpha subunit	HNR49_001572	1.88	3.81 × 10^−8^
A0A841HAT9	Fe-S cluster assembly ATP-binding protein	HNR49_001256	1.75	0.0137
B9LUN1	Cytochrome P450	Hlac_2727	1.51	1.38 × 10^−3^
B9LRJ2	FAD-linked oxidase domain protein	Hlac_0210	1.92	8.03 × 10^−6^
A0A841H9N0	NhaP-type Na^+^/H^+^ or K^+^/H^+^ antiporter	HNR49_000572	1.72	1.26 × 10^−4^

^*a*^ Shown are genes encoding electron transport proteins that are not *c*-type cytochromes and whose protein abundances differed significantly between *H. volcanii* cells grown with lactate as the electron donor under Fe(III)-oxide-reducing conditions and those grown under O_2_-respiring conditions. Fold change was calculated relative to the O_2_ condition [Fe(III)/O_2_]. Proteins were considered significantly different when *P* ≤ 0.05.

From the perspective of cellular energy metabolism, ATPase synthase, a core enzyme complex, converts the electron chemical potential energy of protons (H^+^) into the chemical energy of ATP, thereby sustaining cellular energy metabolism (54). Under Fe(III)-reducing conditions, the relative abundances of V-type ATP synthase subunits (A0A841H9T5 and A0A841HA16) increased by 62.1% and 70.8%, respectively. In addition, the NhaP-type reverse transporter protein (A0A841H9N0), which is responsible for balancing intra- and extracellular Na^+^/H^+^ and thus maintaining proton dynamics to ensure the synchronized transfer of protons and electrons, also showed a 72.2% increase in normalized relative abundance. Together, the upregulation of these energy- and ion-transport-related proteins suggests an enhanced requirement for proton motive force and ion balance during Fe(III)-dependent extracellular electron transfer in *H. volcanii*.

## DISCUSSION

This study demonstrates that under high-salinity anaerobic conditions, *H. volcanii* utilizes its extracellular electron transfer capability to reduce ferrihydrite while maintaining fundamental physiological metabolic activity throughout the process. Based on the lactate and pyruvate levels measured in the preceding experiments, the electron utilization efficiency of dissimilatory iron reduction by *H. volcanii* appears relatively low. Specifically, most of the consumed lactate was not converted into pyruvate; approximately 80% was likely assimilated or completely oxidized into CO_2_. This also indicates that not all electrons derived from substrate oxidation were transferred extracellularly; a considerable portion was consumed to maintain cellular metabolic activity. As an extreme halophile, *H. volcanii* thrives in environments with very high osmotic pressure, which demands significant energy expenditure for maintaining high intracellular K^+^ concentrations, exporting Na^+^, and sustaining transmembrane proton gradients that drive ATP synthesis and nutrient transport (55, 56). Continuous metabolic processes occur inside the cell even under non-growing conditions, requiring reducing power [e.g., NAD(P)H] and energy for protein turnover, damage repair, and basal metabolism (57–59). These activities consume part of the electrons generated from lactate oxidation. In addition, our work revealed that membrane-binding quinoline *b*-type cytochromes may play a crucial role in the direct EET process, while riboflavin contributes to the indirect reduction of extracellular Fe(III) minerals. Nevertheless, the mechanistic role of riboflavin in the reduction of extracellular Fe(III) minerals mediated by *H. volcanii* remains unresolved and is likely multifaceted. Building on the evolving view of flavin-mediated EET, at least three scenarios merit consideration. First, riboflavin may function as a diffusible redox mediator, in which oxidized riboflavin is reduced at (or near) the cell surface and then diffuses to ferrihydrite or an electrode to deliver electrons before returning to the cell in its oxidized form, thereby sustaining a catalytic redox cycle (60). Second, riboflavin may predominantly act through cell-associated, surface-bound flavin cycling, as supported in *Shewanella,* where secreted flavins specifically interact with cell-surface redox proteins (e.g., OmcA/MtrC), behave as protein-associated cofactors, and undergo one-electron cycling that can markedly accelerate electron transfer to insoluble acceptors (60). Third, given that *H. volcanii* is enveloped by an S-layer that shapes the physicochemical interface with the environment, riboflavin could access and participate in redox cycling with S-layer/membrane-associated oxidoreductases, while a fraction may also appear extracellularly via release or exchange processes. Additionally, genome annotations suggest the presence of flavin-dependent, quinone-linked dehydrogenases, and by analogy to the proposed NAD^+^-independent, flavin-dependent D-lactate dehydrogenase/glycolate oxidase in *Methanosarcina acetivorans*, riboflavin could stimulate lactate oxidation and intracellular electron flux, thereby indirectly increasing electron supply to the EET chain (50).

Proteomic analysis showed that the abundance of NADH: quinone oxidoreductase (NDH) protein increased by 60.9% in cells utilizing Fe(III) oxides as electron acceptors compared with O_2_. NADH: quinone oxidoreductase oxidizes NADH to NAD^+^ and transfers electrons to quinones (e.g., ubiquinone UQ), generating the reduced quinol pool (UQH_2_). UQH_2_ can subsequently donate electrons to cytochrome *c* or other electron-transporting proteins that relay electrons toward the extracellular environment (61). As an entry point of the intracellular electron transport chain, the increased abundance of NDH under Fe(III)-reducing conditions is consistent with a greater requirement for intracellular reducing equivalents to be funneled into downstream electron transfer processes associated with EET.

Previous results mentioned that monoheme cytochrome *c* subunit II (B9LN21) is highly expressed in anaerobically cultured cells. Subunit 2 (CO II) of this protein has two adjacent transmembrane regions at its N-terminal end and contains a Cu(A) center, which is capable of receiving electrons from other cytochrome *c* molecules on the outer membrane surface and transferring them to a low-spin heme center (62). Bacterial MHCs play a critical role in the direct reduction of Fe(III) oxides (15, 16, 63). For example, the outer membrane monoheme *c*-type cytochrome of *Geobacter sulfurreducens* functions as a sensor that regulates MHC expression (64, 65). Moreover, genome sequencing of the archaeon *Geoglobus ahangari* revealed 19 putative genes encoding MHCs, several of which are predicted to be localized at the cell surface (25). Thus, the COX2 protein of *H. volcanii* may play a similar role in the transfer of electrons to Fe(III) oxides. B9LN21 may play a potentially important role in the EET process and is a key candidate protein.

Previous studies have shown that *Ferroglobus placidus* encodes three putative menaquinones: iron cytochrome-*c* oxidizing reductases, named Cbc4, which likely serve as the primary electron transport interface for the oxidation of organic compounds in the cytoplasm (21). In the present study, *H. volcanii* was found to possess menaquinol-cytochrome-*c* reductases (B9LS12, B9LTW6). Accordingly, electrons carried by menaquinones may be transferred to periplasmic *c*-type cytochromes in both bacteria and archaea. Upon reaching the cell membrane, membrane-associated quinol-cytochrome oxidoreductase complex cytochrome *b* (QCRI, A0A841HB82; and QCR II, A0A841HBL7) begins to function. This membrane-associated cytochrome *b*, similar in function to menaquinone cytochrome *c* reductase, may also be involved in electron transfer across the cytoplasmic membrane, although further experimental verification is required.

**Fig 8:**
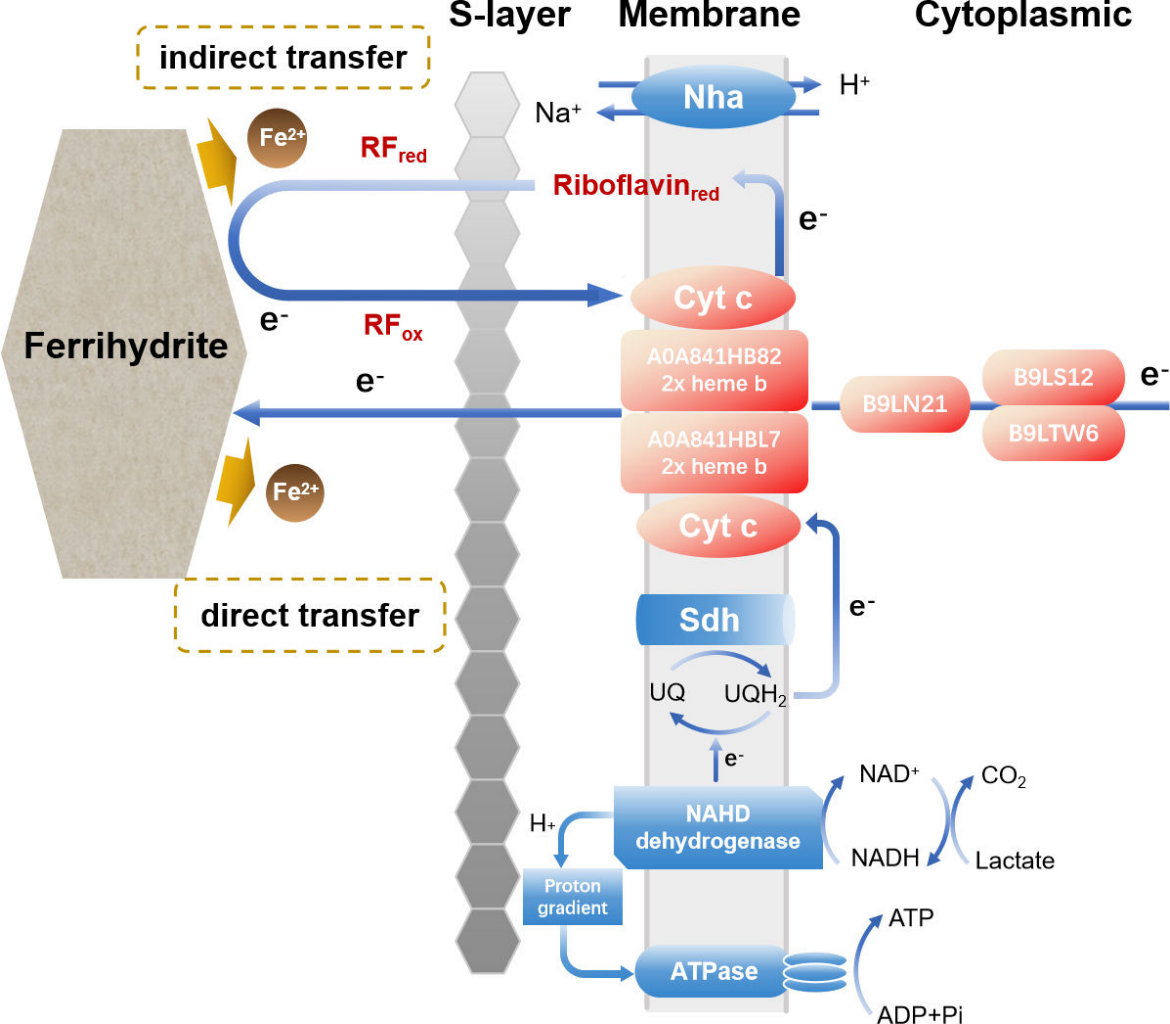
Proposed schematic of extracellular electron transport pathways in *H. volcanii*.

The extracellular electron transport pathways of microorganisms are closely linked to their membrane structure. However, the membrane structure of the extreme halophile *H. volcanii* is very special, which possesses a single cytoplasmic membrane composed of archaeal ether lipids and an external proteinaceous S-layer that functions as the cell wall, but it lacks an outer membrane and a classical periplasmic space. Compared with bacteria, the organization and functional characterization of *H. volcanii* membranes remain less well understood. Based on the electrochemical, chromatographic, and proteomic data, we proposed a putative EET pathway for *H. volcanii*, as illustrated in Fig. 8. Menaquinol-cytochrome-*c* reductases located in the cytoplasm may serve as the primary electron transfer interface for the oxidation of organic matter. NADH transfers electrons to ubiquinone (UQ) via NADH dehydrogenase, which reduces ubiquinone to ubiquinol (UQH_2_). Then the electrons are transferred to cytochrome *c* and quinoline *b*-type cytochrome oxidases. Together, these enzymes and cytochromes may form a continuous electron transfer chain that channels electrons toward extracellular Fe(III) oxides, enabling their reduction. In addition, riboflavin can accept electrons from *c*-type cytochromes, undergoing reduction in the process, and may mediate electron transfer in *H. volcanii* through the three mechanisms discussed earlier. However, since *H. volcanii* possesses a non-conductive S-layer impermeable to minerals, the mechanism by which electrons traverse this barrier to reach the extracellular space remains unclear. Elucidating this process likely requires a suite of redox-active proteins and cofactors that reduce extracellular electron acceptors. Further insight into this mechanism will depend on physiologically relevant functional studies using genetically tractable model systems.

Current research indicates that Fe(III)-reducing archaea employ multiheme *c*-type cytochromes together with quinone/quinol pools as core components of their electron-transport pathways (66). In addition, these archaeas utilize a distinct set of redox-active proteins and cofactors, including molybdopterin oxidoreductases, heterodisulfide reductase, and methanophenazine (67, 68). Traditionally, flavins and flavin-binding proteins have been regarded as features unique to bacterial Fe(III) reduction. For instance, the thermophilic archaea *Geoglobus ahangari*, *Geoglobus acetivorans*, and *Ferroglobus placidus* are unable to reduce Fe(III) (oxyhydr)oxides encapsulated in alginate beads (21, 23, 69), suggesting the absence of a secreted electron shuttle or chelating agent for electron transfer. In contrast, this work demonstrates that the extreme halophile  *H. volcanii* can secrete riboflavin as an extracellular electron shuttle, mediating indirect electron transfer to Fe(III) oxides. This finding, therefore, implies that archaea and bacteria may share similar redox-active molecules and protein systems for Fe(III) reduction, pointing to possible evolutionary convergence in their EET pathways.

While bacterial Fe(III) oxide reduction has been extensively studied, research on the molecular mechanisms for archaeal Fe(III) reduction remains limited and largely preliminary. This knowledge gap is primarily attributable to the scarcity of genetically tractable archaeal model systems. In this context, *H. volcanii* exhibits high genomic stability and well-established genetic tools, rendering it a promising emerging model archaeon for elucidating EET mechanisms. These findings advance our understanding of biogeochemical iron cycling in hypersaline ecosystems, highlighting the previously underestimated role of archaea in mediating metal reduction processes in extreme environments. Moreover, the identification of EET capabilities in extreme halophile *H. volcanii* suggests potential applications in the electrochemical treatment of pollutants in hypersaline wastewater.

## Data Availability

Data will be made available on request. The mass spectrometry proteomics data have been deposited to the ProteomeXchange Consortium (https://proteomecentral.proteomexchange.org) via the iProX partner repository (38, 39) with the data set identifier PXD074223. All other data supporting the findings of this study are available within the paper.
